# Community characteristics that attract physicians in Japan: a cross-sectional analysis of community demographic and economic factors

**DOI:** 10.1186/1478-4491-7-12

**Published:** 2009-02-18

**Authors:** Masatoshi Matsumoto, Kazuo Inoue, Satomi Noguchi, Satoshi Toyokawa, Eiji Kajii

**Affiliations:** 1 Division of Community and Family Medicine, Centre for Community Medicine, Jichi Medical University, Tochigi, Japan; 2 Department of Public Health, Graduate School of Medicine, University of Tokyo, Tokyo, Japan

## Abstract

**Background:**

In many countries, there is a surplus of physicians in some communities and a shortage in others. Population size is known to be correlated with the number of physicians in a community, and is conventionally considered to represent the power of communities to attract physicians. However, associations between other demographic/economic variables and the number of physicians in a community have not been fully evaluated. This study seeks other parameters that correlate with the physician population and show which characteristics of a community determine its "attractiveness" to physicians.

**Methods:**

Associations between the number of physicians and selected demographic/economic/life-related variables of all of Japan's 3132 municipalities were examined. In order to exclude the confounding effect of community size, correlations between the physician-to-population ratio and other variable-to-population ratios or variable-to-area ratios were evaluated with simple correlation and multiple regression analyses. The equity of physician distribution against each variable was evaluated by the orenz curve and Gini index.

**Results:**

Among the 21 variables selected, the service industry workers-to-population ratio (0.543), commercial land price (0.527), sales of goods per person (0.472), and daytime population density (0.451) were better correlated with the physician-to-population ratio than was population density (0.409). Multiple regression analysis showed that the service industry worker-to-population ratio, the daytime population density, and the elderly rate were each independently correlated with the physician-to-population ratio (standardized regression coefficient 0.393, 0.355, 0.089 respectively; each p < 0.001). Equity of physician distribution was higher against service industry population (Gini index = 0.26) and daytime population (0.28) than against population (0.33).

**Conclusion:**

Daytime population and service industry population in a municipality are better parameters of community attractiveness to physicians than population. Because attractiveness is supposed to consist of medical demand and the amenities of urban life, the two parameters may represent the amount of medical demand and/or the extent of urban amenities of the community more precisely than population does. The conventional demand-supply analysis based solely on population as the demand parameter may overestimate the inequity of the physician distribution among communities.

## Background

Physicians are one of the most essential human resources for maintaining health. Equal distribution of physicians in consideration of health care needs is a crucial part of health policy. However, in reality, the unequal distribution of physicians is a serious problem in many countries. Physicians are disproportionately concentrated in cities and are in short supply in rural areas [1-4]. Especially in Japan, where medical practice is financially based on a fee-for-service reimbursement system and there is no restriction on practice location, physician distribution is determined largely by the market and by physicians' individual preferences. As a consequence, physicians are highly concentrated in communities that are financially and geographically attractive to them, which results in their so-called maldistribution.

The maldistribution itself is not problematic. If attractiveness is equal to medical demand, maldistribution should be justified because the concentration of physicians in high-need communities is a proper allocation of this limited human resource. However, in many societies, there is a gap between the distribution of needs and the distribution of physicians, hence, the shortage of physicians in rural areas is a serious problem.

The power of communities to attract physicians consists of two elements: the amount of medical demand and the extent of urban amenities [1,5-8]. Medical demand is composed of factors such as population size, elderly rate and morbidity rate. Thus, it is difficult to pinpoint the amount of medical demand in a community [6,9-11].

However, evaluating medical demand and representing it quantitatively is necessary in order to arrive at an accurate assessment of resources and subsequently a desirable distribution of health resources. Therefore, population size is conventionally used for representing the quantity of medical needs. The physician-to-population ratio is used to evaluate the demand-supply balance of physicians in a community [1,5,7,12].

The municipality (i.e. city, town or village) is the smallest administrative unit, and is the most often used geographical unit for communities in Japan, while the county is the comparable unit in countries such as the United States of America. Municipalities with higher physician-to-population ratios are regarded as areas of physician oversupply and municipalities with lower ratios are judged as areas of physician shortage [12]. The municipality- or county-level physician-to-population ratio has also been used to examine the longitudinal change of the demand-supply balance in a single area [1,5,7].

The second element of community attractiveness, the amenities of urban life, can also have a substantial impact on physician distribution, and this factor explains why physicians are overconcentrated in some areas. Physicians tend to prefer living in urban areas, so the distribution of physicians is biased toward urban areas [1,13,14]. This urban preference is probably due to the highly biased birth origin of physicians toward urban areas [13,15,16].

The urban amenities that physicians tend to prefer are also a complex concept that is difficult to quantify. Past studies have revealed that the extent of urban amenities correlates well with population size. The physician-to-population ratio is known to increase proportionately with population [1,5]. This means that physicians would rather practise in cities with large populations despite the intense competition for survival in such areas [1,5].

Population is thus a parameter of both medical demand and urban amenities. This indicates that the population can represent the whole range of attractiveness of a community. However, it is unknown whether the population alone is the best parameter of the community attractiveness among many available variables. There are no studies that have evaluated the many potential associations between demographic or economic variables and the number of physicians in a community. If we can evaluate the physician-pulling power of communities by means of multiple variables, we can identify more precisely what constitutes "medical demand" and "urban amenities". These types of data can clarify our understanding of the equal distribution of physicians.

In this study, we examine the strength of associations between demographic, economic or life-related variables of municipalities and the number of physicians in the municipalities by using a widely available dataset of Japan's 3132 municipalities. Variables that have independent correlations with the number of physicians are regarded as potential parameters of the community attractiveness. We also calculate the equity of physician distribution against each of the possible parameters of attractiveness in order to re-evaluate the maldistribution of physicians, as indicated by conventional physician-to-population analysis.

## Methods

Japan has three levels of government: municipal, prefectural and national. Municipalities (cities, towns and villages) are the basic geographical units of administration. Prefectures and municipalities in Japan are roughly comparable to states and counties in the United States, respectively. All the data analysed in this study are from *Statistical Observations of the Municipalities 2005*, which includes 100 variables related to demographics, natural environment, economic activities, labour force, health and security in each of Japan's 3132 municipalities [17]. The dataset was produced by the Ministry of Internal Affairs and Communications, and was available online free of charge. The number of physicians is one of the variables included in the dataset, but it was transferred from the National Physician Census, where all practising physicians in Japan are obliged to register their work addresses every two years. Each of the variables selected for analysis is described in Table 1.

**Table 1:** Municipality variables selected for analysis

**Variable**	**Explanation**
Population	Number of registered residents

Daytime population	[Population] + [number of commuters from outside] - [number of commuters to outside]

Commuters from outside	Population of other municipalities who commute to the municipality

Commuters to outside	Population of the municipality who commute out

Foreigners	Population who are not Japanese

Elderly population	Population who are 65 years old or older

Elderly rate	Proportion of those who are 65 years old or older among the population

Workers	Number of workers

Primary industry workers	Number of workers who engage in agriculture/fishery/mining industry

Sales of primary industry products	Total annual sales of the agriculture/fishery/mining products (yen)

Manufacturing industry workers	Number of workers who engage in manufacturing industry

Sales of manufactured products	Total annual sales of manufactured products (yen)

Service industry workers	Number of service industry workers (excluding health care workers)

Sales of commercial goods	Total annual sales of commercial goods (yen)

Executives	Number of executives of companies and public organizations

Total jobless rate	Proportion of those who cannot find a job among employable population

Total income of residents	Total of annual incomes of all residents (yen)

Residential land price	Price of residencial land per square kilometre (yen)

Commercial land price	Price of commercial land per square kilometre (yen)

Divorces	Number of divorces per year

Crimes	Number of crime cases per year

Area	Total area (square kilometres)

Length of paved roads	Total length of paved roads (kilometres)

The simplest way to identify which variables could represent community attractiveness is to examine simple correlation coefficients between the number of physicians and selected variables of municipalities, assuming that the distribution of physicians is influenced by the attractiveness of the municipalities in which they live. The correlation analysis, however, has one problem. Because the values of most of the variables (including the number of physicians) depend on the size of the population or the size of the area of the municipality, most of the variables correlate well with the number of physicians, whether they represent attractiveness or not. For example, the number of kindergartens in a municipality correlates well with its number of physicians, not because the number of kindergartens represents the community's attractiveness, but because both values are dependent on and confounded by the size of the municipality.

Thus, we need to examine the correlations from which the influences of the population size and area size have been excluded. For this purpose, we calculated the number of physicians per 100 000 residents in each of the municipalities, and examined simple correlation coefficients between the physician-to-population ratio and other variable-to-population ratios.

For population and daytime population, variable-to-area ratios were used instead of variable-to-population ratios because these variables directly represent population. If the number of physicians distributes equally against population, the physician-to-population ratios of municipalities should remain constant. However, in reality, physician-to-population ratios vary substantially from one area to another [1].

The variability of physician-to-population ratios indicates that population, the conventional parameter supposed to reflect medical demand, does not necessarily represent the full spectrum of an area's pulling power (attractiveness). Hence, we regarded variables where the ratio to population or ratio to area correlated well with the physician-to-population ratio as possible parameters representing a portion of attractiveness that cannot be represented by population. Because most of the variables in the dataset were not normally distributed, all correlations were presented with Spearman's rho correlation.

Next, we extracted variables in which the ratio to population or ratio to area showed relatively strong correlations with the physician-to-population ratio. We also extracted additional variables that are theoretically associated with medical demand (i.e. proportion of the elderly, income level of residents).

We then conducted multiple-regression analysis in which the extracted variables were treated as explanatory variables and the physician-to-population ratio as the outcome variable. This analysis was conducted to reveal the extent to which each variable-to-population ratio or variable-to-area ratio independently correlated with the physician-to-population ratio, and how much the fluctuation in total of the variables could predict the fluctuation of the physician-to-population ratio among the municipalities.

In this multivariate analysis, all the variables except for the proportion of the aged were log_10_-transformed because they were not normally distributed. The variance inflation factor (VIF) of each explanatory variable, which is an index that measures how much the variance of a coefficient is increased due to collinearity, was calculated to examine the severity of multicollinearity.

Population size and other variables in which the ratio to population or to area showed stronger correlations with the number of physicians in the multiple regression analysis were regarded as possible parameters of community attractiveness, and the equity of physician distribution against each of the parameters was evaluated by the Lorenz curve and Gini index. Both measures have traditionally been used to show the extent of income equity among the members of a society, but they are also used for the analysis of equity of physician distribution against population [1,18].

For example, in the analysis of physician-population distribution we first ranked all the municipalities by physician-to-population ratio. Next, we calculated both the cumulative proportion of physicians and that of population of each municipality in ascending order of the physician-to-population ratio.

We then plotted all the points representing the municipalities onto the plane of coordinates with the X-axis representing the cumulative proportion of population and the Y-axis representing that of physicians [1]. The plotted line is the Lorenz curve and the diagonal line between (0,0) point and (1,1) point represents the complete equity in the physician-population distribution. The degree of the arc of the Lorenz curve corresponds to the degree of inequity.

Finally, we calculated the Gini index, which is the area between the Lorenz curve and the complete equity line, divided by the triangle under the equity line. The Gini index ranges from 0 to 1; the smaller the value, the more equal the distribution.

The same procedures were used in evaluating equity of physician distribution against the other possible parameters of attractiveness. We regarded variables against which physicians are more equally distributed as better parameters of community attractiveness: that is, better parameters of medical demand and/or urban amenities.

All the statistical analyses were carried out using SPSS^® ^for Windows, version 11.5 (SPSS Inc., Japan). The analyses were two-tailed, and P < 0.05 was considered statistically significant.

## Results

The results of simple correlation analysis between the physician-to-population ratio and other selected variables divided by population or area size are shown in Table 2. The service industry workers-to-population ratio showed the strongest correlation with the physician-to-population ratio (R = 0.543). Other variables such as commercial land price, sales of goods per person and daytime population density were also positively correlated (R = 0.527, 0.472, 0.451, respectively). In short, density of commercial activity and people correlated best with the physician-to-population ratio. Some variables such as manufacturing industry workers/population ratio were negatively correlated, suggesting that these variables have a negative impact on the number of physicians when the effect of municipality size is adjusted for.

**Table 2:** Basic characteristics of community variables and their correlations with physician-to-population ratio (n = 3,132)

**Variables of municipalities**	**Mean**	**IQR**	Correlation*	P**
Service industry workers/unit population	26 004	18 639 – 28 979	0.543	<0.001

Commercial land price	80 645	27 525 – 88 175	0.527	<0.001

Sales of commercial goods/person	2 003 300	618 200 – 1 653 200	0.472	<0.001

Daytime population density	1 020	245 – 854	0.451	<0.001

Residential land price	39 208	11 600 – 47 150	0.436	<0.001

Population density	1 018	271 – 914	0.409	<0.001

Workers/unit population	96 194	95 408 – 97 225	0.364	<0.001

Executives/unit population	4 176	3 146 – 5 030	0.349	<0.001

Crimes/unit population	1 313	755 – 1 688	0.326	<0.001

Income/person	3 117 434	2 816 800 – 3 293 300	0.298	<0.001

Total jobless rate	3.8	2.8 – 4.6	0.252	<0.001

Commuters from outside/unit population	151	83 – 173	0.232	<0.001

Length of paved roads/unit area	0.7	0.4 – 0.9	0.225	<0.001

Foreigners/unit population	536	162 – 639	0.222	<0.001

Divorces/unit population	182	134 – 226	0.196	<0.001

Sales of manufactured products/unit population	199 912	44 266 – 229 916	0.112	<0.001

Manufacturing industry workers/unit population	13 905	9 392 – 16 788	-0.049	0.005

Commuters to outside/unit population	189	120 – 258	-0.105	<0.001

Elderly rate	24.0	18.7 – 28.5	-0.226	<0.001

Sales of primary industry products/unit population	21 910	5 474 – 27 284	-0.319	<0.001

Primary industry workers/unit population	7 426	2 732 – 10 678	-0.381	<0.001

Outcome variable is the number of physicians per 100 000 residents (mean 126; IQR 53–155)

*Spearman's rho correlation coefficient

**Probability of coefficient being zero

IQR: Interquartile range

Unit population is 100,000 residents

Unit area is a square kilometer

The results of multiple regression analysis in which the outcome variable is the physician-to-population ratio are shown in Table 3. As explanatory variables, two variables that showed higher correlations with physician-to-population ratio were used: service industry workers/population ratio and the daytime population density. Other variables with higher correlations, such as population density and sales of goods per person, showed strong collinearity with one or both of the two variables, and therefore were not used in the regression model. Two other variables, the proportion of those aged 65 or older among the population (elderly rate) and the average income per person, were also used as explanatory variables because they are theoretically expected to influence medical demand.

**Table 3:** Multiple regression analysis between physician-to-population ratio and other variables of municipalities (n = 3,132)

	Coefficient*	P**	**VIF**
Daytime population density	0.355	<0.001	1.826

Elderly rate	0.089	<0.001	1.747

Income per person	0.010	0.578	1.588

Service industry workers per unit population	0.393	<0.001	1.146

Multiple correlation coefficient (R)	0.564	<0.001	

R^2^	0.318		

Outcome variable is the number of physicians per 100 000 residents

*Standard partial correlation coefficient

**Probability of coefficient being zero

VIF: variance inflation factor

All variables except for elderly rate were log10-transformed

The square of the multiple correlation coefficient (R^2^) of the model was 0.318; that is, the fluctuations of explanatory variables in total explain 32% of the fluctuation of physician-to-population ratio among the municipalities. The service industry workers/population ratio, the daytime population density and elderly rate were each independently correlated with the physician-to-population ratio (standardized regression coefficient [B] = 0.393, 0.355, 0.089, respectively; each p < 0.001). The average income per person was not significantly correlated (B = 0.010, p = 0.578). No strong collinearity was seen among the explanatory variables (each variance inflation factor [VIF]<2).

**Figure 1:**
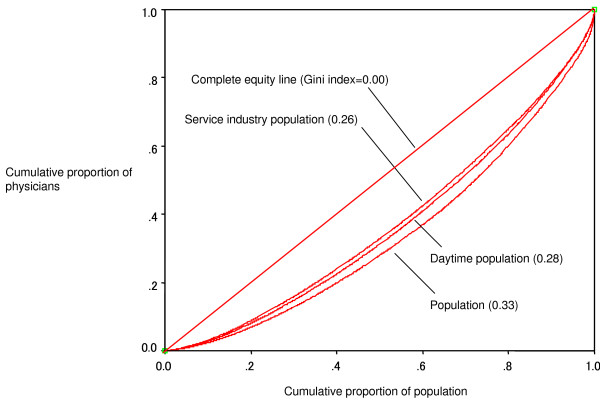
**Lorenz curves and Gini indices of physician distribution against populations**.

Figure 1 shows the Lorenz curves and Gini indices of physician distribution against three indicators assumed to represent community attractiveness: population, daytime population and service industry population. The figure indicates that physicians are most equally distributed against the service industry population (Gini index = 0.26), next, against the daytime population (0.28) and least equally, against the general population (0.33).

## Discussion

This study demonstrated that the variation of the conventional indicator of physician demand-supply balance, the physician-to-population ratio among municipalities was to a substantial degree explained by variations in the daytime population density, service industry worker-to-population ratio, and elderly rate among municipalities. Daytime population density and service industry worker-to-population ratio were independently and strongly correlated with the physician-to-population ratio. When population, daytime population and population of service industry workers were each regarded as parameters of community attractiveness, the distribution of physicians was least equal against population and was most equal against service industry workers.

In Japan, there is no legal restriction on practice location of physicians. Physicians can freely choose their places of practice. All medical practices in Japan are covered by the National Health Insurance System and profit financially from its fee-for-service reimbursement system [19]. Physician distribution in Japan therefore tends to be driven by the market, and by physicians' own preferences for urban location. In this context, the community attractiveness, that is, the community's pulling power for physicians is determined largely by the amount of medical demand and extent of urban amenities of the community. Population size is usually seen as the parameter that best reflects the amount of medical demand [1]. This is an assumption upon which the physician-to-population ratio as an indicator of the demand/supply balance of physicians is based. Japanese health policies, particularly those on physician supply and rural health, have been created based on the physician-to-population analysis of areas [14].

However, the results of this study support a hypothesis that daytime population is an even better indicator of the community attractiveness (demand and/or urbanity) than population. Daytime population density correlated with the physician-to-population ratio slightly better than population density. Variation of daytime population density among communities was independently correlated with variation of physician-to-population ratio, indicating that there are areas in which physicians appear to be over- or undersupplied against the registered (nighttime) population, but, in fact, adequately supplied against daytime population. Theoretically speaking, rather than the mere number of registered residents, the total number of residents who remain in the area during the daytime and who commute from the outside (i.e. daytime population) should be a more accurate reflection of medical needs.

In Japan, most medical offices close at 6:00 pm. It was reported that the proportion of hospitals open for non-emergency outpatients after 6:00 pm on weekdays was 10.3% to 12.4%, and the proportion of clinics was 13.7% to 26.3% [20]. Thus, most medical services are provided during business hours.

The data of Hong et al. support our reported results. Based on the population data of United States counties, they reported that areas in which higher proportions of the residents commuted out tend to have lower physician-to-population ratios, suggesting that daytime population is a better indicator of a community's medical demand than nighttime population [21].

The strongest correlation of physician population with service industry population among community variables indicates that the service industry population best represents a community's attractiveness. This indirectly supports another hypothesis that "accessible daytime population" and commercial activity are good indicators of medical demand and/or urbanity.

"Accessible daytime population" is defined as the sum of the population and persons who are outside the community but can potentially gain access to the community in a relatively short time during the day. The "accessible daytime population" should theoretically be a better indicator of medical needs than population or even daytime population. It includes not only the population existing in the area but also people in the neighborhood communities who have a strong connection to the area in terms of transportation and/or commercial activity.

Theoretically the population of service industry workers in the area could represent the size of the "accessible daytime population". Service industry workers provide a variety of goods and services to their customers. The potential customers of the service industry workers are not only the residents of the area, but also people who can gain access to the area from outside. The areas with high service industry workers/population ratios are expected to be commercial centres of their wider regions, and be strongly connected to surrounding areas via infrastructure such as roads and railways.

The potential customers of commercial centres are thus residents of wider regions. From the perspective of hospital and clinic administration, the concentration of physicians in such commercial centres is to be expected, because they have access to a larger potential customer population and hence can possibly reap larger profits.

Moreover, the commercial centres should be more "urban" than areas of similar population size that experience less commercial activity. From the perspective of physicians' own preferences, such urban areas with commerce would be desirable places to practise and live. In contrast, areas with low service industry workers-to-population ratios are expected to be isolated areas in which the sizes of accessible daytime populations are almost equal to the sizes of their registered populations. The populations of potential patients (i.e. the amount of medical demand) in such areas should be smaller than in communities of comparable size with commercial activity. Regardless of the amount of medical demand, however, such rural areas would not be attractive to physicians, most of whom have urban origins and therefore a preference for urban life.

The more equal distribution of physicians against daytime population and service industry population than that observed against population itself also supports the hypothesis that these variables are better indicators of community attractiveness than population. The result also suggests that maldistribution of physicians can be overestimated when the distribution analysis is based solely on the conventional physician-to-population ratio.

If we assume that daytime population and service industry population are more sensitive parameters of medical demand than population, taking into account distribution analysis of physicians against these parameters enables us to obtain a clearer sense of the demand/supply balance of physicians in communities, and to ascertain what their equal distribution actually is. These types of data can lead to more appropriate public policy actions.

The extent to which population, daytime population and service industry population represent medical demand is still largely unknown. As shown in the results, these variables are parameters of community attractiveness. Attractiveness, however, is not necessarily the same as demand. In particular, the plausibility of service industry population as an indicator of medical demand is unclear.

Population and daytime population can be considered as direct indicators of medical needs because health service users are included in each of the populations. The service industry population, however, does not necessarily overlap with the user population; it is an indirect, surrogate parameter that is expected to represent the size of population that has access to the area.

As mentioned above, community attractiveness in Japan is largely determined by demand and urban amenities. Service industry population thus might represent the extent of urban amenities more than does demand. If this is the case, equal distribution of physicians against service industry population is neither desirable nor justifiable because concentration of physicians in urban areas beyond the amount of demand in the areas is not a rational allocation of the limited human resource. It is thus necessary to first demonstrate, before concluding that the parameter represents medical needs, that the parameter correlates well with the real and directly measured accessible population.

We employed the municipality as the geographical unit for the analysis. An assumption is required for the municipality variables to be an indicator of community attractiveness. The assumption is that health-seeking behaviour of patients and the provision of health services take place within the boundary of municipality.

It has been reported that this assumption does not necessarily hold true [21,22]. A substantial proportion of patients cross county borders to visit their physicians [21,22]. Past studies in the United States revealed that the percentage of physician visits that involve county-border crossing ranges from 7% to 47% according to the type of the county; the rate was lowest in large metropolitan counties and highest in rural counties adjacent to metropolitan counties [22,23]. It might thus be problematic to use the county (in the case of the United States) and municipality (in the case of Japan) as the geographical unit for the analysis of physician supply.

Although several alternative units have been proposed and tested [23-25], these new analytical tools are much less available to researchers and policy-makers than the municipality/county-based data, so their usage is quite limited. In practical terms, it is most convenient to use municipality-based data because of the high availability and accuracy of the data, particularly in terms of demographics and health care variables. Moreover, municipality-based analysis of physician supply is useful for policy-making because the municipality is both an administrative and a geographical unit [12].

For these reasons, municipality-based data are still widely used to analyse the distribution of physicians, regardless of their limitations. The results of this study revealed that there are variables in widely available municipality data that can potentially take into account the cross-border effect and represent with greater accuracy the community medical demand and/or urban amenities.

## Conclusion

Population size is a parameter that represents a community's attractiveness to physicians, and the conventional physician-to-population ratio is a relatively valid way of to analyse the demand-supply balance of physicians. However, daytime population and service industry population in a municipality represent the attractiveness of an area better than population does, and thus each can be a better parameter of medical demand and/or urban amenities of the area. The number of people in the area during daytime and the volume of commercial activity may be key components of medical demand in and/or the urban amenities of an area. By adding these new parameters to the conventional analysis that uses population, we may be able to evaluate the equity of physician distribution more precisely.

## Competing interests

The authors declare that they have no competing interests.

## Authors' contributions

MM directed the study, contributed to design, methods and manuscript writing. EK, KI, ST and SN contributed to design, statistical analysis and manuscript writing.

## References

[B1] Kobayashi Y, Takaki H (1992). Geographic distribution of physicians in Japan. Lancet.

[B2] Australian Medical Workforce Advisory Committee (2000). The General Practice Workforce in Australia.

[B3] Bureau of Health Professions (1992). Rural Health Professions Facts: Supply and Distribution of Health Professions in Rural America.

[B4] Rivo ML, Kindig DA (1996). A report card on the physician work force in the United States. N Engl J Med.

[B5] Rosenthal MB, Zaslavsky A, Newhouse JP (2005). The geographic distribution of physicians revisited. Health Serv Res.

[B6] Cooper RA, Getzen TE, Laud P (2003). Economic expansion is a major determinant of physician supply and utilization. Health Serv Res.

[B7] Schwartz WB, Newhouse JP, Bennett BW, Williams AP (1980). The changing geographic distribution of board-certified physicians. N Engl J Med.

[B8] Ernst RL, Yett DE (1985). Physician location and specialty choice.

[B9] Smaje C, Grand JL (1997). Ethnicity, equity and the use of health services in the British NHS. Soc Sci Med.

[B10] Morris S, Sutton M, Gravelle H (2005). Inequity and inequality in the use of health care in England: an empirical investigation. Soc Sci Med.

[B11] van Doorslaer E, Masseria C, Koolman X (2006). Inequalities in access to medical care by income in developed countries. CMAJ.

[B12] Ministry of Health, Labour and Welfare The working party report on national supply of physicians (Ishi no jukyu ni kansuru kentoukai houkokusyo).

[B13] Matsumoto M, Okayama M, Inoue K, Kajii E (2005). Factors associated with rural doctors' intention to continue a rural career: a survey of 3072 doctors in Japan. Aust J Rural Health.

[B14] Jichi Medical School (2002). Chiiki-iryo hakusyo (The white paper on community healthcare).

[B15] Matsumoto M, Inoue K, Kajii E (2008). Characteristics of medical students with rural origin: Implications for selective admission policies. Health Policy.

[B16] Takayashiki A, Okayama M, Mise J, Kajii E (2003). Igakusei no syusshinchi to syourai no hekichikinmu kibou. (The birthplaces of medical students and their intentions of rural practice). Primary Care.

[B17] Statistic Bureau, Ministry of Internal Affairs and Communications (2005). Toukei de miru shikuchouson no sugata (Statistical observations of the municipalities) Electronic ed.

[B18] Brown MC (1994). Using Gini-style indices to evaluate the spatial patterns of health practitioners: theoretical considerations and an application based on Alberta data. Soc Sci Med.

[B19] Ikegami N, Campbell JC (1996). The art of balance in health policy: maintaining Japan's low-cost, egalitarian system Japanese edition.

[B20] Ministry of Health LaW (2007). Iryo-sisetsu chosa 2007 (Health Facility Census 2007).

[B21] Hong W, Kindig DA (1992). The relationship between commuting patterns and health resources in nonmetropolitan counties of the United States. Med Care.

[B22] Kleinman JC, Makuc D (1983). Travel for ambulatory medical care. Med Care.

[B23] Makuc D, Kleinman JC, Pierre MB (1985). Service areas for ambulatory medical care. Health Serv Res.

[B24] Makuc DM, Haglund B, Ingram DD, Kleinman JC, Feldman JJ (1991). The use of health service areas for measuring provider availability. J Rural Health.

[B25] Goodman DC, Mick SS, Bott D, Stukel T, Chang CH, Marth N, Poage J, Carretta HJ (2003). Primary care service areas: a new tool for the evaluation of primary care services. Health Serv Res.

